# Mortality and Life-Sustaining Therapy Decisions in Patients With Cancer and Acute Respiratory Failure Due to COVID-19 or Other Causes: An Observational Study

**DOI:** 10.3389/fmed.2021.620818

**Published:** 2021-05-03

**Authors:** Renato Scarsi Testa, Ana Paula Agnolon Praça, Antonio Paulo Nassar Junior, Pauliane Vieira Santana, Valdelis Novis Okamoto, Ramon Teixeira Costa, Pedro Caruso

**Affiliations:** ^1^Intensive Care Unit, Antonio Cândido (AC) Camargo Cancer Center, São Paulo, Brazil; ^2^Pulmonary Division, Heart Institute (InCor), Hospital das Clínicas da Faculdade de Medicina da Universidade de São Paulo, São Paulo, Brazil

**Keywords:** cancer, respiratory insufficiency, COVID-19, intensive care unit, critical care outcomes

## Abstract

It is unknown if patients with cancer and acute respiratory failure due to COVID-19 have different clinical or cancer-related characteristics, decisions to forgo life-sustaining therapies (LST), and mortality compared to patients with cancer and acute respiratory failure due to other causes. In a cohort study, we tested the hypothesis that COVID-19 was associated with increased in-hospital mortality and decreased decisions to forgo LST in patients with cancer and acute respiratory failure. We employed two multivariate logistic regression models. Propensity score matching was employed as sensitivity analysis. We compared 382 patients without COVID-19 with 65 with COVID-19. Patients with COVID-19 had better performance status, less metastatic tumors, and progressive cancer. In-hospital mortality of patients with COVID-19 was lower compared with patients without COVID-19 (46.2 vs. 74.6%; *p* < 0.01). However, the cause of acute respiratory failure (COVID-19 or other causes) was not associated with increased in-hospital mortality [adjusted odds ratio (OR) 1.27 (0.55–2.93; 95% confidence interval, CI)] in the adjusted model. The percentage of patients with a decision to forgo LST was lower in patients with COVID-19 (15.4 vs. 36.1%; *p* = 0.01). However, COVID-19 was not associated with decisions to forgo LST [adjusted OR 1.21 (0.44–3.28; 95% CI)] in the adjusted model. The sensitivity analysis confirmed the primary analysis. In conclusion, COVID-19 was not associated with increased in-hospital mortality or decreased decisions to forgo LST in patients with cancer and acute respiratory failure. These patients had better performance status, less progressive cancer, less metastatic tumors, and less organ dysfunctions upon intensive care unit (ICU) admission than patients with acute respiratory failure due to other causes.

## Introduction

Intensive care unit (ICU) admissions, acute respiratory failure (ARF), and poor outcomes are more common in patients with COVID-19 and cancer than in patients with COVID-19 without cancer (1, 2). Furthermore, patients with cancer are also susceptible to ARF due to other causes (3). It is unknown if patients with cancer and ARF due to COVID-19 (COVID-19 ARF) have different clinical characteristics, cancer-related characteristics, and in-hospital mortality compared to patients with cancer and ARF due to other causes (non-COVID-19 ARF).

Severe COVID-19 in patients with cancer could increase decisions to forgo life-sustaining therapies (LST) because COVID-19 is perceived as a high-mortality disease. By contrast, COVID-19 acute presentation and the worldwide commotion to save patients with COVID-19 might decrease the decision to forgo LST. COVID-19 impact on the decision to forgo LST is unknown in patients with cancer.

We tested the hypothesis that COVID-19 was associated with increased in-hospital mortality and decreased decisions to forgo LST in patients with cancer and ARF. We also compared clinical and cancer-related characteristics between cancer patients with COVID-19 ARF and non-COVID-19 ARF.

## Methods

### Study Design and Patients

We designed a cohort study using data collected from a cancer center with 490 beds (AC Camargo Cancer Center, São Paulo, Brazil), with 50 being ICU beds. The sample size calculation demanded, at least, 65 patients with and 195 without COVID-19 ARF (1:3 ratio) (see Supplementary Material).

The study compared a prospective cohort of patients with cancer and COVID-19 ARF with a historical control group of patients with cancer and non-COVID-19 ARF. In both groups, we included all adult patients with solid tumors or hematologic malignancies and unplanned ICU admission due to ARF, and we excluded patients with cancer remission >5 years, decision to forgo LST prior to ICU, and admissions for post-operative care. Patients with COVID-19 were included during the pandemic (March until August 2020), while patients without COVID-19 were included before the pandemic (March until August, in the years 2012 until 2017, respectively). If a patient had multiple ICU admissions, only the first was considered.

Data were collected and maintained in a structured electronic spreadsheet designated to the present study. In the hospital, COVID-19 was confirmed by a positive SARS-CoV-2 RT-PCR in a patient with compatible symptoms or image of COVID-19.

According to Brazilian regulations, the forgoing of LST requires a consensual decision of the patient (or a next of kin) and the attending team. In our ICU, the forgoing of life-sustaining therapies requires a consensual decision of intensivists, oncologists, and patients (or a next of kin).

The local ethics committee approved this study (2521/18L) and waived the need for informed consent.

### Data Collection

Upon ICU admission, patient's demographic characteristics, Simplified Acute Physiology Score (SAPS 3) (4); Eastern Cooperative Oncology Group (ECOG) performance status (5); the Sequential Organ Failure Assessment Score (SOFA) and the respiratory parameters of the SOFA score (respiratory SOFA) (6); Charlson Comorbidity Index (7); specific comorbidities [arterial hypertension, diabetes, chronic pulmonary disease (chronic obstructive pulmonary disease or chronic restrictive pulmonary disease), heart diseases (chronic arrhythmia needing treatment or systolic or diastolic heart failure), overweight or obesity (body mass index > 25 kg/m^2^)]; type of cancer (non-metastatic solid tumor, metastatic solid tumor, or hematologic malignancies); recent systemic cancer treatment (chemotherapy or immunotherapy in the last month); site of the solid tumors; and response to cancer treatment (newly diagnosed without treatment, partial or complete response, or progressive cancer despite treatment) were recorded.

During the ICU stay, the use of invasive mechanical ventilation (>24 h), the use of non-invasive mechanical ventilation, the use of vasopressors (defined as any use of noradrenaline, dobutamine, vasopressin, or adrenaline), the need of hemodialysis, and any decision to forgo life-sustaining therapies (withholding or withdrawing of treatment) were recorded. According to Brazilian regulations, the forgoing of life-sustaining therapies requires a consensual decision of the patient (or a next of kin) and the attending team. In our hospital, the forgoing of life-sustaining therapies requires a consensual decision of intensivists and oncologists.

Finally, the in-hospital mortality was recorded.

### Statistical Analysis

Categorical and continuous data were presented as percentages and median [25–75% interquartile range (IQR)] values, respectively. Categorical variables were compared using the chi-square test or Fisher's exact-test, as appropriate. Continuous variables were compared with the Mann–Whitney-test.

To test the hypothesis that COVID-19 was associated with increased in-hospital mortality and decreased decisions to forgo LST in patients with cancer and ARF, we employed two multivariate logistic regression models. We used a directed acyclic graph to identify confounders (8), and the following confounders were included in the both models: age, sex, type of cancer, response to cancer treatment, ECOG, Charlson Comorbidity Index, and the ARF cause (COVID-19 or non-COVID-19) (Supplementary Figures 1, 2).

As a sensitivity analysis, we employed propensity score matching, with balance checking (absolute standardized mean difference), to match COVID-19 ARF to non-COVID-19 ARF patients (9).

We depicted (Kaplan–Meier) and compared (log-rank-test) the 28-day mortality curves of patients with COVID-19 ARF and non-COVID-19 ARF.

Statistical analyses were performed by SPSS software (Version 23.0. Armonk, NY: IBM Corp). *P*-values ≤ 0.05 were considered significant. We followed the recommendations of the STROBE statement that guides the report of observational studies (10).

## Results

During the pre-pandemic period, we included all 382 patients with non-COVID-19 ARF. During the pandemic, 107 patients with confirmed COVID-19 diagnosis were admitted to the ICU and 65 patients were included. Forty patients were excluded because they were admitted to post-operative care, 19 patients had cancer remission >5 years, and three patients had readmissions.

### Clinical and Cancer-Related Characteristics

Patients with COVID-19 ARF had better performance status, less metastatic tumors, and progressive cancer. They had lower Charlson Comorbidity Index but more overweight/obesity. Upon ICU admission, patients with COVID-19 ARF had less severe acute organ dysfunctions. However, during ICU stay, they needed more life-sustaining therapies and had longer ICU and hospital lengths of stay than patients with non-COVID-19 ARF (Table 1). Among the hospital survivors, the hospital length of stay of the patients with COVID-19 [24 days (16–42)] was higher than the patients without COVID-19 [12 days (7–19)] (*p* < 0.01).

**Table 1:** Characteristics of patients with cancer and acute respiratory failure due to COVID-19 and non-COVID-19 causes.

**Variable**	**Non-COVID-19**	**COVID-19**	***p***
	**(*n* = 382)**	**(*n* = 65)**	
**Age (years)**	64 (56–74)	62 (55–70)	0.26
**Male**	199 (52.1)	37 (56.9)	0.50
**Charlson comorbidity index**	7 (6–9)	4 (2–6)	<0.01
**Comorbidities**			
Hypertension	150 (39.3)	32 (49.2)	0.13
Diabetes	61 (16.0)	17 (26.2)	0.05
Chronic pulmonary disease	46 (12.0)	9 (13.8)	0.68
Cardiovascular disease	37 (9.7)	8 (12.3)	0.50
BMI > 25 kg/m^2^	144 (38.4)	41 (63.1)	<0.01
**ECOG performance status**			<0.01
0–1	102 (26.7)	47 (72.3)	
2–4	280 (73.3)	18 (27.7)	
**Cancer type**			<0.01
Non-metastatic solid	86 (22.5)	31 (47.7)	
Metastatic solid	247 (64.7)	17 (26.2)	
Hematologic malignancies	49 (12.8)	17 (26.2)	
**Solid tumor site**			0.05
Breast	52 (15.6)	12 (25.0)	
Lung	83 (24.9)	5 (10.4)	
Prostate	10 (3.0)	6 (12.5)	
Head and neck	25 (7.5)	2 (4.2)	
Colorectal	38 (11.4)	6 (12.5)	
Pancreas	8 (2.4)	3 (6.3)	
Other	117 (35.1)	14 (29.2)	
**Response to treatment**			<0.01
Newly diagnosed	41 (10.7)	5 (7.7)	
Complete or partial	138 (36.1)	39 (60.0)	
Progressive disease	203 (53.1)	21 (32.3)	
Cancer treatment			
**Chemotherapy last month**	205 (53.7)	18 (27.7)	<0.01
**Immunotherapy last month**	13 (3.4)	3 (3.9)	0.28
**Bone marrow transplant**	12 (3.1)	4 (6.2)	0.26
**SAPS3 at ICU admission**	69 (62–77)	58 (49–70)	<0.01
**SOFA at ICU admission**	5 (4–7)	3 (1–4)	<0.01
**Respiratory SOFA at ICU admission**	3 (2–3)	3 (2–3)	0.52
**During ICU stay**			
Invasive MV	141 (36.9)	38 (58.5)	0.02
Non-invasive MV	142 (37.2)	19 (29.2)	0.26
Vasopressors	63 (16.5)	37 (56.9)	<0.01
Hemodialysis	32 (8.4)	18 (27.7)	<0.01
**ICU length of stay**	4 (2–7)	9 (3–18)	<0.01
**ICU mortality**	196 (51.3)	27 (41.5)	0.17
**Decision to forgo LST**	138 (36.1)	10 (15.4)	0.01
**Hospital length of stay**	7 (3–14)	22 (13–35)	<0.01
**In-hospital mortality**	285 (74.6)	30 (46.2)	<0.01

*ICU, intensive care unit; SAPS 3, Simplified Acute Physiology Score; SOFA, Sequential Organ Failure Assessment Score; Respiratory SOFA, the value of respiratory parameter of the SOFA score; BMI, body mass index; ECOG, Eastern Cooperative Oncology Group; LST, life-sustaining therapies; MV, mechanical ventilation; Invasive MV, invasive mechanical ventilation for more than 24 h. Chronic pulmonary disease is chronic obstructive pulmonary disease or chronic restrictive pulmonary disease; heart diseases are chronic arrhythmia needing treatment and systolic or diastolic heart failure; vasopressors are defined as any use of noradrenaline, vasopressin or adrenaline. Categorical and continuous data are presented as absolute counts (percentages) and median (25–75% interquartile range), respectively. Categorical variables were compared using the chi-square test or Fisher's exact-test, as appropriate. Continuous variables were compared with the Mann–Whitney-test*.

### In-Hospital Mortality

In-hospital mortality of patients with COVID-19 ARF was lower compared with patients with non-COVID-19 ARF (46.2 vs. 74.6%; *p* < 0.01) [unadjusted odds ratio 0.29 (0.17–0.50; 95% confidence interval, CI)] (Table 1 and Supplementary Figure 3). However, adjusting for age, sex, type of cancer, response to cancer treatment, ECOG, Charlson Comorbidity Index, and the ARF cause (COVID-19 or non-COVID-19), COVID-19 as the cause of ARF was not associated with increased in-hospital mortality [adjusted odds ratio 1.27 (0.55–2.93; 95% CI)] (Table 2).

**Table 2:** Multivariate analysis for in-hospital mortality and decisions to forgo life-sustaining therapies of critically ill patients with cancer admitted to ICU with acute respiratory failure.

**Variable**	**Survivors**	**Non-survivors**	^*^Adjusted OR	**Forgo LST**	**Not forgo LST**	^*^Adjusted OR
	**(*n* = 315)**	**(*n* = 132)**		**(*n* = 148)**	**(*n* = 299)**	
**Age**	64 (56–73)	64 (55–73)	1.97 (0.99–3.62)	64 (56–73)	64 (56–72)	1.01 (0.98–1.04)
**Male**	176 (55.8)	60 (45.5)	0.97 (0.94–1.00)	79 (53.4)	157 (52.5)	0.86 (0.50–1.49)
**Charlson comorbidity index**	7 (6–9)	6 (4–8)	0.87 (0.71–1.07)	7 (6–9)	6 (4–8)	0.96 (0.78–1.18)
**ECOG performance status**						
0–1	44 (14.0)	105 (79.5)	REF	19 (12.8)	130 (43.5)	REF
2–4	271 (86.0)	27 (20.5)	47.48 (22.17–101.69)	129 (87.2)	169 (56.5)	5.08 (2.57–10.07)
**Cancer type**						
Non-metastatic solid tumor	53 (16.8)	64 (48.5)	REF	22 (14.9)	95 (31.8)	REF
Metastatic solid tumor	218 (69.2)	46 (34.8)	7.37 (2.69–20.17)	117 (79.1)	147 (49.2)	2.19 (0.83–5.78)
Hematological malignancies	44 (14.0)	22 (16.7)	3.59 (1.49–8.63)	9 (6.1)	57 (19.1)	0.64 (0.22–1.87)
**Response to cancer treatment**						
Newly diagnosed	15 (11.4)	31 (9.8)	REF	1 (0.7)	45 (15.1)	REF
Complete or partial response	79 (59.8)	98 (31.1)	0.87 (0.34–2.23)	8 (5.4)	169 (56.5)	2.35 (0.28–19.67)
Progressive disease	38 (28.8)	186 (59.0)	2.56 (0.95–6.92)	139 (93.9)	85 (28.4)	73.63 (9.75–555.77)
**COVID-19 as cause of ARF**	30 (9.5)	35 (26.5)	1.27 (0.55–2.93)	10 (6.8)	55 (18.4)	1.21 (0.44–3.28)

*ARF, acute respiratory failure; SOFA, Sequential Organ Failure Assessment Score; ECOG, Eastern Cooperative Oncology Group; ICU, intensive care unit; LST, life-sustaining therapies; OR, odds ratio. Categorical and continuous data are presented as frequencies (percentages) and median (25–75% interquartile range), respectively*.

* *The confounders included in the model (age, male, Charlson, ECOG, cancer type, and response to cancer treatment) serve exclusively to control for confounding. The observed associations between these confounders and the outcome (in-hospital mortality or decision to forgo life-sustaining therapies) have not been subject to the same control of confounding as the exposure (COVID-19). Therefore, residual confounding and other biases often heavily influence these associations*.

### Decision to Forgo Life-Sustaining Therapies

The percentage of patients with a decision to forgo LST was lower in patients with COVID-19 ARF than in patients with non-COVID-19 ARF (15.4 vs. 36.1%; *p* = 0.01) [unadjusted odds ratio 0.32 (0.16–0.65; 95% CI)] (Table 1). However, adjusting for age, sex, type of cancer, response to cancer treatment, ECOG, Charlson Comorbidity Index, and the ARF cause (COVID-19 or non-COVID-19), COVID-19 as the cause of ARF was not associated with decisions to forgo LST [adjusted odds ratio 1.21 (0.44–3.28; 95% CI)] (Table 2).

### Sensitivity Analyses

As the primary analyses, sensitivity analysis also showed that COVID-19 was neither associated with in-hospital mortality nor with decision to forgo LST (Table 3).

**Table 3:** Comparison of patients with cancer and COVID-19 acute respiratory failure with matched patients with non-COVID-19 acute respiratory failure.

**Variable**	^*^Matched Non-COVID-19	**COVID-19**	***p***	**ASMD**
	**(*n* = 50)**	**(*n* = 50)**		
**Age (years)**	61 (48–69)	60 (54–68)	0.93	0.00
**Male**	27 (54.0)	25 (50.0)	0.84	0.08
**Charlson comorbidity index**	5 (3–6)	6 (3–6)	0.64	0.07
**Comorbidities**				
Arterial hypertension	15 (30.0)	24 (48.0)	0.99	
Diabetes	5 (10.0)	13 (26.0)	0.07	
Chronic pulmonary disease	6 (12.0)	6 (12.0)	0.99	
Cardiovascular disease	2 (4.0)	6 (12.0)	0.27	
BMI > 25 kg/m^2^	19 (38.0)	29 (58.0)	0.07	
**ECOG performance status**			0.84	0.08
0–1	31 (62.0)	33 (66.0)		
2–4	19 (38.0)	17 (34.0)		
**Cancer type**			0.71	0.12
Non-metastatic tumor	26 (52.0)	22 (44.0)		
Metastatic tumor	13 (26.0)	16 (32.0)		
Hematologic malignancies	11 (22.0)	12 (24.0)		
**Solid tumor site**			0.24	
Breast	9 (23.1)	10 (26.3)		
Lung	9 (23.1)	4 (10.5)		
Prostate	1 (2.6)	4 (10.5)		
Head and neck	5 (12.8)	1 (2.6)		
Colorectal	5 (12.8)	4 (10.5)		
Pancreas	1 (2.6)	3 (7.9)		
Other	9 (23.1)	12 (31.6)		
**Cancer treatment response**			0.94	0.03
Newly diagnosed	5 (10.0)	4 (8.0)		
Complete or partial	27 (54.0)	28 (56.0)		
Progressive disease	18 (36.0)	18 (36.0)		
**Chemotherapy last month**	24 (48.0)	16 (32.0)	0.15	
**Immunotherapy last month**	1 (3.0)	2 (6.0)	0.62	
**Bone marrow transplant**	4 (8.0)	4 (8.0)	1.00	
**SAPS3 at ICU admission**	67 (56–76)	58 (48–74)	0.05	
**SOFA at ICU admission**	4 (1–6)	3 (1–5)	0.52	0.06
**Respiratory SOFA at ICU admission**	3 (2–3)	3 (2–3)	0.22	
**During ICU stay**				
Invasive MV	19 (38.0)	28 (56.0)	0.11	
Non-invasive MV	23 (46.0)	13 (26.0)	0.06	
Vasopressors	8 (16.0)	28 (56.0)	<0.01	
Hemodialysis	4 (8.0)	15 (30.0)	<0.01	
**ICU length of stay**	5 (3–11)	7 (4–18)	0.08	
**ICU mortality**	17 (34.0)	21 (42.0)	0.54	
**Decision to forgo LST**	11 (22.0)	8 (16.0)	0.61	
**Hospital length of stay**	10 (6–18)	22 (13–35)	<0.01	
**In-hospital mortality**	22 (44.0)	23 (46.0)	0.99	

*ASMD, absolute standardized mean difference; ICU, intensive care unit; SAPS 3, Simplified Acute Physiology Score; SOFA, Sequential Organ Failure Assessment Score; BMI, body mass index; ECOG, Eastern Cooperative Oncology Group; LST, life-sustaining therapies; MV, mechanical ventilation. Respiratory SOFA is the value of respiratory parameter of the SOFA score; chronic pulmonary disease is chronic obstructive pulmonary disease or chronic restrictive pulmonary disease; cardiovascular diseases are chronic arrhythmia needing treatment and systolic or diastolic heart failure; invasive MV is invasive mechanical ventilation for more than 24 h; vasopressors are defined as any use of noradrenaline, vasopressin, or adrenaline*.

*Categorical and continuous data are presented as absolute counts (percentages) and median (25–75% interquartile range), respectively. Categorical variables were compared using the chi-square test or Fisher's exact-test, as appropriate. Continuous variables were compared with the Mann–Whitney-test*.

* *Patients with COVID-19 and non-COVID-19 acute respiratory failure were matched for age, sex, type of cancer, response to cancer treatment, SOFA, ECOG, and Charlson Comorbidity Index. The propensity score was calculated using logistic regression and pairs were matched by the nearest neighbor with a caliper distance ≤ 0.05*.

## Discussion

Patients with cancer and COVID-19 ARF had different cancer-related and clinical characteristics from their non-COVID-19 counterparts, such as better performance status and less progressive cancer. These differences probably occurred because patients with poor performance status and progressive cancer had low mobility and were less exposed to COVID-19. Additionally, patients with a high probability of survival might be preferentially admitted to the ICU, as part of the effort to improve ICU resource allocation during the pandemic.

Upon ICU admission, patients with COVID-19 ARF had less severe organ dysfunctions than patients with non-COVID-19 ARF; however, during ICU stay, they needed more invasive mechanical ventilation, vasopressors, and hemodialysis. These results probably occurred because at presentation, severe COVID-19 is predominantly a respiratory disease; however, its typically long course led to progressive clinical deterioration and increased use of life-sustaining therapies (11, 12). Confirming the long COVID-19 course, in our study, patients with COVID-19 ARF had a significantly longer ICU and hospital lengths of stay than patients with non-COVID-19 ARF.

The observed clinical and cancer-related differences explain the lower mortality found in patients with cancer and COVID-19 ARF because the severity of organ dysfunctions upon ICU admission (13, 14), poor performance status (13, 14), progressive cancer (14), hematologic malignancies, and metastatic tumors (15) are associated with in-hospital mortality of critically ill patients with cancer.

Patients with COVID-19 required more hemodialysis, probably due to a direct impact of COVID-19 on the kidney, because the standard of care was similar between groups. Patients without COVID-19 presented a higher percentage of lung cancer than patients with COVID-19, probably reflecting a direct thoracic cancer involvement as a cause of ARF in patients without COVID-19.

It has been shown that poor performance status and progressive cancer are associated with more decisions to forgo LST (16), while hematological malignancies was associated with less decisions to forgo LST (17). In the present study, patients with COVID-19 ARF had better performance status, less progressive cancer, and more hematological malignancies compared to non-COVID-19 patients. These differences probably determined the lower percentage of decisions to forgo LST in patients with COVID-19 ARF.

The present study has limitations. It was conducted at a single dedicated cancer center and physicians must carefully evaluate the results of single-center trials within the context of their clinical experience and the preferences of their patients to determine how best to translate research to the bedside (18). The causes of ARF in non-COVID-19 patients were unknown for several patients, and some causes probably were non-infectious, such as cancer spread and idiopathic alveolar hemorrhage. However, only 5 to 20% of ARF causes are non-infectious (19–21), around 10% of patients have more than one cause (19), and even with the best efforts ~20% of causes are impossible to be established in patients with cancer (20, 21). We did an extensive characterization of lung injury and clinical status upon ICU admission, but some relevant variables were not recorded, such as previous thoracic radiation therapy, presence of pulmonary and pleural metastasis to characterize lung injury, and absolute neutrophil count and hemoglobin level to characterize the patient upon ICU admission. Finally, patients with non-COVID-19 ARF were included during a 6-year period (2012 until 2017) while patients with COVID-19 were included in 2020, and improvements in overall ICU care along this period (22) should be considered in interpreting the results.

In conclusion, COVID-19 was not associated with increased in-hospital mortality or decreased decisions to forgo life-sustaining therapies in patients with cancer and acute respiratory failure. Patients with cancer and COVID-19 acute respiratory failure had better performance status, less progressive cancer, less metastatic tumors, and less organ dysfunctions upon ICU admission than patients with non-COVID-19 acute respiratory failure.

## Data Availability Statement

The raw data supporting the conclusions of this article will be made available by the authors, without undue reservation.

## Ethics Statement

The studies involving human participants were reviewed and approved by AC Camargo Cancer Center Ethics Committee (2521/18L). Written informed consent for participation was not required for this study in accordance with the national legislation and the institutional requirements.

## Author Contributions

RT, AP, and PC made substantial contributions to the conception of the work, acquisition, and interpretation of data. ANJ and RC made substantial contributions to the design of the study and interpretation of data. PS and VO made substantial contributions to acquisition and interpretation of data. PC drafted the manuscript. All authors revised the manuscript critically for important intellectual content and gave final approval of the version to be published.

## Conflict of Interest

The authors declare that the research was conducted in the absence of any commercial or financial relationships that could be construed as a potential conflict of interest.

## References

[1] Giannakoulis VG Papoutsi E Siempos II. Effect of cancer on clinical outcomes of patients with COVID-19: a meta-analysis of patient data. JCO Glob Oncol. (2020) 6:799–808. 10.1200/GO.20.00225 32511066 PMC7328119

[2] Liang W Guan W Chen R Wang W. Cancer patients in SARS-CoV-2 infection: a nationwide analysis in China. Lancet Oncol. (2020) 21:335–7. 10.1016/S1470-2045(20)30096-6 32066541 PMC7159000

[3] Azoulay E Mokart D Kouatchet A Demoule A Lemiale V. Acute respiratory failure in immunocompromised adults. Lancet Respir Med. (2019) 7:173–86. 10.1016/S2213-2600(18)30345-X 30529232 PMC7185453

[4] Moreno RP Metnitz PGH Almeida E Jordan B Bauer P Campos RA. SAPS 3—from evaluation of the patient to evaluation of the intensive care unit. Part 2: Development of a prognostic model for hospital mortality at ICU admission. Intensive Care Med. (2005) 31:1345–55. 10.1007/s00134-005-2763-5 16132892 PMC1315315

[5] Oken MM Creech RH Tormey DC Horton J Davis TE McFadden ET. Toxicity and response criteria of the Eastern Cooperative Oncology Group. Am J Clin Oncol. (1982) 5:649–56. 10.1097/00000421-198212000-00014 7165009

[6] Vincent JL Moreno R Takala J Willatts S De Mendonça A Bruining H. The SOFA (Sepsis-related Organ Failure Assessment) score to describe organ dysfunction/failure. On behalf of the Working Group on Sepsis-Related Problems of the European Society of Intensive Care Medicine. Intensive Care Med. (1996) 22:707–10. 10.1007/BF01709751 8844239

[7] Charlson ME Pompei P Ales KL MacKenzie CR. A new method of classifying prognostic comorbidity in longitudinal studies: development and validation. J Chronic Dis. (1987) 40:373–83. 10.1016/0021-9681(87)90171-8 3558716

[8] Lederer DJ Bell SC Branson RD Chalmers JD Marshall R Maslove DM. Control of confounding and reporting of results in causal inference studies. Ann Am Thorac Soc. (2019) 16:22–8. 10.1513/AnnalsATS.201808-564PS 30230362

[9] Austin PC. An introduction to propensity score methods for reducing the effects of confounding in observational studies. Multivariate Behav Res. (2011) 46:399–424. 10.1080/00273171.2011.568786 21818162 PMC3144483

[10] von Elm E Altman DG Egger M Pocock SJ Gøtzsche PC Vandenbroucke JP. The strengthening the reporting of observational studies in epidemiology (STROBE) statement: guidelines for reporting observational studies. J Clin Epidemiol. (2008) 61:344–9. 10.1016/j.jclinepi.2007.11.008 18313558

[11] Zhou F Yu T Du R Fan G Liu Y Liu Z. Clinical course and risk factors for mortality of adult inpatients with COVID-19 in Wuhan, China: a retrospective cohort study. Lancet. (2020) 395:1054–62. 10.1016/S0140-6736(20)30566-3 32171076 PMC7270627

[12] Zheng X Yang H Li X Li H Xu L Yu Q. Prevalence of kidney injury and associations with critical illness and death in patients with COVID-19. Clin J Am Soc Nephrol. (2020) 15:1549–56. 10.2215/CJN.04780420 32943396 PMC7646240

[13] Soares M Salluh JIF Spector N Rocco JR. Characteristics and outcomes of cancer patients requiring mechanical ventilatory support for >24 hrs. Crit Care Med. (2005) 33:520–6. 10.1097/01.CCM.0000155783.46747.04 15753742

[14] Azevedo LCP Caruso P Silva UVA Torelly AP Silva E Rezende E. Outcomes for patients with cancer admitted to the ICU requiring ventilatory support results from a prospective multicenter study. Chest. (2014) 146:257–66. 10.1378/chest.13-1870 24480886

[15] Dai M Liu D Liu M Zhou F Li G Chen Z. Patients with cancer appear more vulnerable to SARS-CoV-2: a multicenter study during the COVID-19 outbreak. Cancer Discov. (2020) 10:783–91. 10.1158/2159-8290.CD-20-0422 32345594 PMC7309152

[16] Lautrette A Garrouste-Orgeas M Bertrand PM Goldgran-Toledano D Jamali S Laurent V. Respective impact of no escalation of treatment, withholding and withdrawal of life-sustaining treatment on ICU patients' prognosis: a multicenter study of the Outcomerea Research Group. Intensive Care Med. (2015) 41:1763–72. 10.1007/s00134-015-3944-5 26149302

[17] da Cruz VM Camalionte L Caruso P. Factors associated with futile end-of-life intensive care in a cancer hospital. Am J Hosp Palliat Med. (2015) 32:329–34. 10.1177/1049909113518269 24399608

[18] Levy MM. Facilitating knowledge transfer with single-center trials. Crit Care Med. (2009) 37:3120–3. 10.1097/CCM.0b013e3181bdd9ae 19789436

[19] Azoulay E Pickkers P Soares M Perner A Rello J Bauer PR. Acute hypoxemic respiratory failure in immunocompromised patients: the Efraim multinational prospective cohort study. Intensive Care Med. (2017) 43:1808–19. 10.1007/s00134-017-4947-1 28948369

[20] Azoulay É Mokart D Lambert J Lemiale V Rabbat A Kouatchet A. Diagnostic strategy for hematology and oncology patients with acute respiratory failure: randomized controlled trial. Am J Respir Crit Care Med. (2010) 182:1038–46. 10.1164/rccm.201001-0018OC 20581167

[21] Wohlfarth P Turki AT Steinmann J Fiedler M Steckel NK Beelen DW. Microbiologic diagnostic workup of acute respiratory failure with pulmonary infiltrates after allogeneic hematopoietic stem cell transplantation: findings in the era of molecular- and biomarker-based assays. Biol Blood Marrow Transplant. (2018) 24:1707–14. 10.1016/j.bbmt.2018.03.007 29550627 PMC7110883

[22] Zampieri FG Romano TG Salluh JIF Taniguchi LU Mendes PV Nassar AP. Trends in clinical profiles, organ support use and outcomes of patients with cancer requiring unplanned ICU admission: a multicenter cohort study. Intensive Care Med. (2021) 47:170–9. 10.1007/s00134-020-06184-2 32770267

