# Supplementary material for: Mortality and Life-Sustaining Therapy Decisions in Patients With Cancer and Acute Respiratory Failure Due to COVID-19 or Other Causes: An Observational Study
Source: Front Med (Lausanne). 2021 May 3;8:620818. doi: 10.3389/fmed.2021.620818 (PMC8126609; doi:10.3389/fmed.2021.620818)
Supplement: Supplementary file 1 [file Data_Sheet_1.PDF]

Supplementary Material

Mortality and life-sustaining therapy decisions in patients with cancer and acute respiratory failure due to Covid-19 or other causes: an observational study

Methods

We estimated the sample size using a formula for observational studies (http://riskcalc.org:3838/samplesize/). The considered type I error was 0.05, the type II error was 0.20 (power 80%), the case to control ratio was 1:3, the in-hospital mortality probability of 45% in the control group (non-Covid-19) and 65% in the Covid-19 group. The in-hospital mortality probability of 45% was based on a prospective multicenter observational study that included our ICU and showed that the in-hospital mortality of patients with cancer requiring ventilatory support was 42% (1). The sample size calculation demanded, at least, 65 patients with Covid-19 and 195 without it (1:3 ratio).

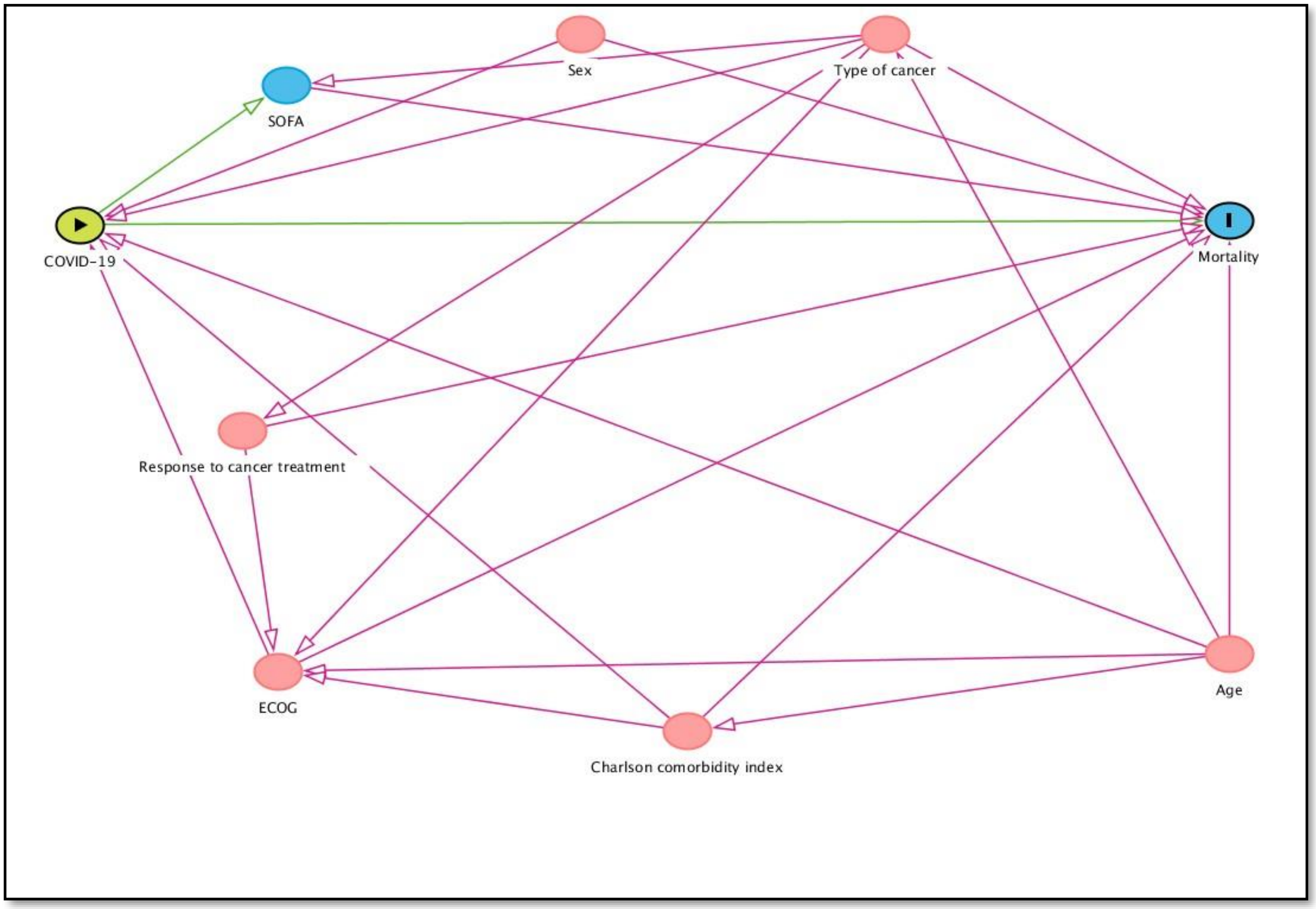

Figure E1. Visual representation of the causal model using directed acyclic graph. Covid-19 is the exposure and in-hospital mortality as the outcome. Age, SOFA score, sex, type of cancer, response to cancer treatment, ECOG, Charlson comorbidity index were included as possible confounders. The model discarded SOFA score as a confounder.

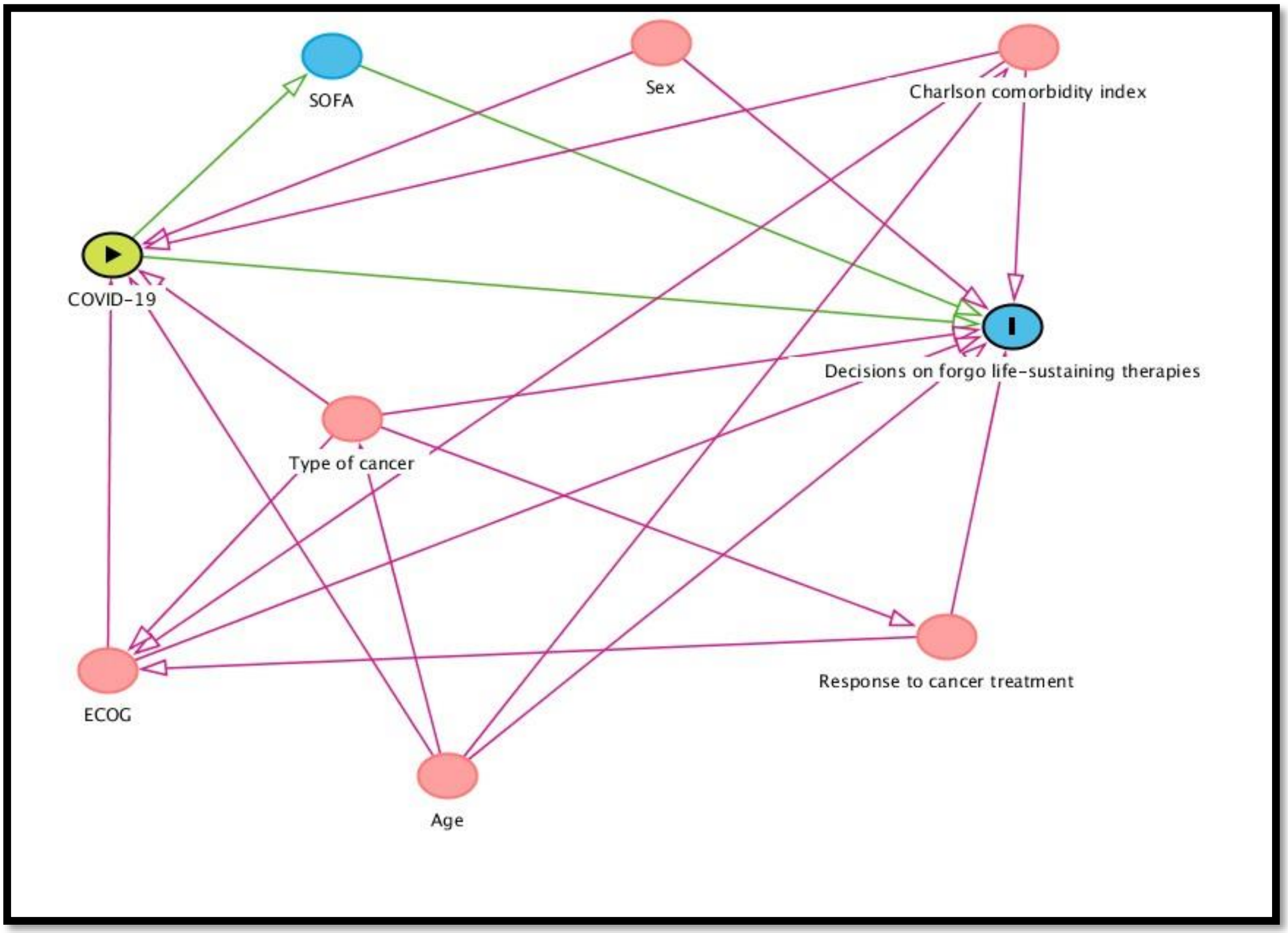

Figure E2. Visual representation of the causal model using directed acyclic graph. Covid-19 is the exposure and decision to forgo life-sustaining therapies as the outcome. Age, SOFA score, sex, type of cancer, response to cancer treatment, ECOG, Charlson comorbidity index were included as possible confounders. The model discarded SOFA score as a confounder.

Results

Figure E3.

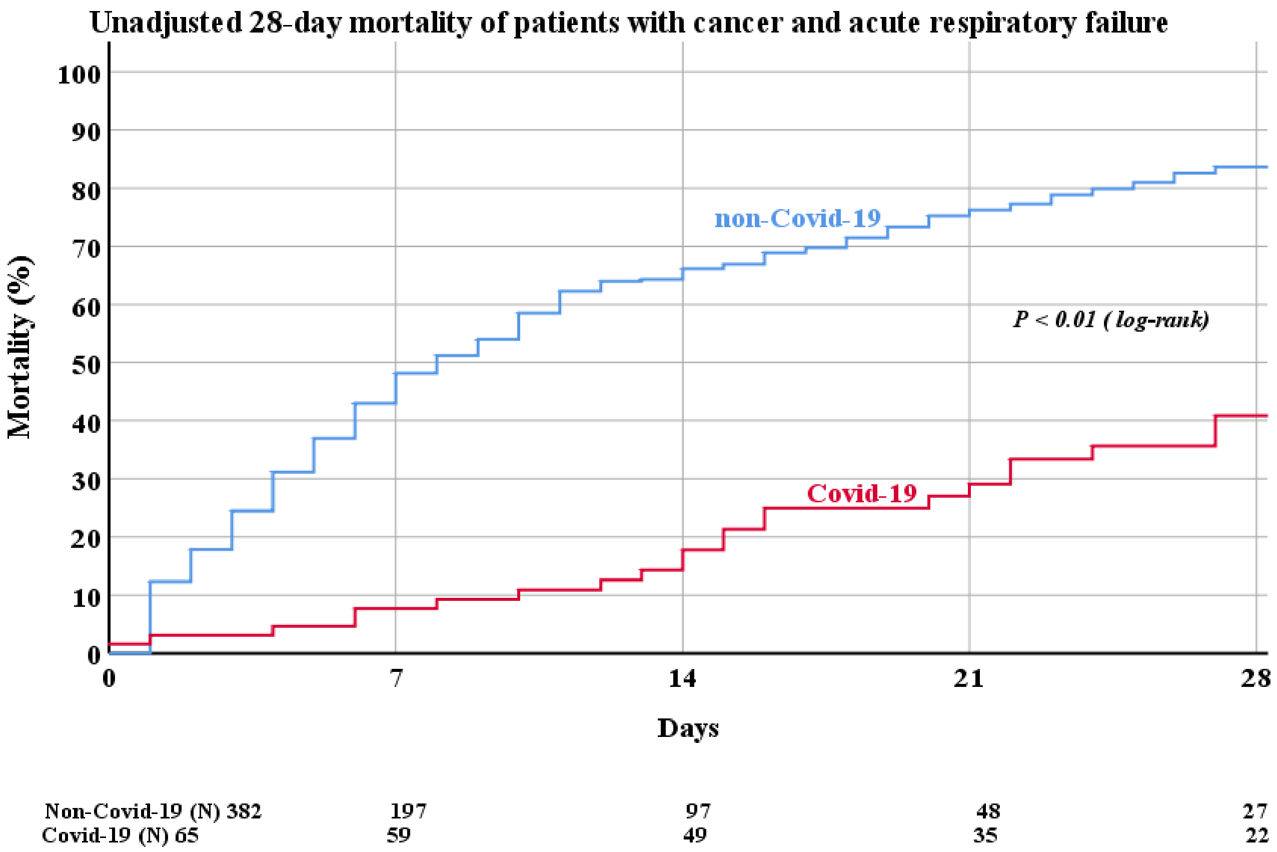

References

1. Azevedo LC, Park M, Salluh JI, Rea-Neto A, Souza-Dantas VC, Varaschin P, Oliveira MC, Tierno PF, dal-Pizzol F, Silva UV, Knibel M, Nassar AP Jr, Alves RA, Ferreira JC, Teixeira C, Rezende V, Martinez A, Luciano PM, Schettino G, Soares M. Clinical outcomes of patients requiring ventilatory support in Brazilian intensive care units: a multicenter, prospective, cohort study. *Crit Care* 2013 4;17:R63.
